# End-of-life care planning and one-year outcomes in intensive care unit patients aged 80 years and older: a single-center cohort study

**DOI:** 10.62675/2965-2774.20260209

**Published:** 2026-01-08

**Authors:** Henrique Mezzomo Pasqual, Maria Doroti Sousa da Rosa, Jonas Michel Wolf, Regis Goulart Rosa, Cassiano Teixeira

**Affiliations:** 1 Hospital Moinhos de Vento Porto Alegre RS Brazil Hospital Moinhos de Vento - Porto Alegre (RS), Brazil; 2 Universidade Federal do Rio Grande do Sul Porto Alegre RS Brazil Universidade Federal do Rio Grande do Sul - Porto Alegre (RS), Brazil; 3 Universidade Federal de Ciências da Saúde de Porto Alegre Porto Alegre RS Brazil Universidade Federal de Ciências da Saúde de Porto Alegre - Porto Alegre (RS), Brazil

The global increase in elderly patients admitted to intensive care units (ICUs) underscores the importance of understanding their outcomes and the role of advance care planning. We conducted a prospective cohort study to evaluate the characteristics, care directives, and one-year outcomes of patients aged ≥ 80 years admitted to a private ICU in southern Brazil.

This study included 62 consecutively admitted patients with a median age of 87 years (IQR 84 - 91), 55% of whom were women. Most admissions were medical (88.7%), and 37.1% of the patients were classified as frail at baseline (assessed using the Clinical Frailty Scale [CFS]). The majority of patients were functionally dependent, and 29.1% were very dependent (Katz Index score ≤ 2) according to the Katz Index. Although 56.4% had an ongoing relationship with their attending physician, only 9.7% had any form of care directive at ICU admission, most of which were discussed during hospitalization after the initiation of life support at ICU admission (Table 1).

**Table 1:** Key characteristics and outcomes of patients aged ≥ 80 years in the intensive care unit

Variable		
Age (years)		87 (84 - 91)
Female sex		34 (55.0)
Medical admission		55 (88.7)
Baseline frailty (CFS ≥5)		23 (37.1)
Katz Index score		
	0 - 1	12 (19.4)
	2 - 3	20 (32.3)
	≤ 2	18 (29.1)
Ongoing care with attending physician		35 (56.4)
Advance directive at ICU admission		6 (9.7)
Treatment limitation during ICU stay		15 (24.2)
MV initiated		22 (35.5)
RRT initiated		5 (8.1)
ICU mortality		18 (29.0)
Hospital mortality		23 (37.0)
1-year mortality		31 (50.0)
Survivors with increased dependency		14 (100.0)
Survivors needing caregivers		10 (71.4)
Survivors with limited activities		11 (78.6)
Survivors with pain/anxiety (EQ-5D-5L)		9 (64.3)

CFS - Clinical Frailty Scale; ICU - intensive care unit; MV – mechanical ventilation; RRT - renal replacement therapy. The results are expressed as medians (interquartile ranges) or n (%).

During their ICU stays, 24.2% of patients had defined care limitations, primarily after the initiation of invasive life support (53.3%). Intensive care unit mortality was 29%, and hospital mortality was 37%. One-year mortality reached 50%. Among the 14 survivors evaluated at one year (via structured telephone interviews conducted by trained clinicians), all had a worsened dependency status, and 71.4% required caregiver support at home. In addition, 78.6% reported significant limitations related to routine activities, while 64.3% described moderate or severe anxiety and pain. Despite receiving aggressive interventions, many survivors reported low quality of life and reduced functional capacity, as assessed using the EQ-5D-5L scale.

This study emphasizes the lack of structured goals-of-care discussions, even among patients with prior medical follow-up. Early and consistent end-of-life discussions could better align interventions with patient preferences and improve the quality of care. The observed high long-term mortality and functional decline highlight the need to integrate geriatric and palliative care approaches within ICU settings. Our findings are consistent with prior research indicating that very elderly people often face limited long-term benefits from aggressive ICU treatments and underscore the importance of early goals-of-care discussions.^(1-4)^ In particular, studies such as the ICE-CUB2 trial and recent European registry data have demonstrated poor long-term outcomes in elderly ICU patients despite high resource utilization.^(5-7)^ Previous authors^(8-10)^ have emphasized the importance of structured family communication in the ICU, as well as barriers that hinder goals-of-care discussions in seriously ill patients, both of which reinforce the urgency of implementing advance care planning strategies.

In conclusion, this study highlights a significant gap in end-of-life care planning among critically ill patients aged ≥ 80 years. These findings should prompt efforts to improve communication, incorporate shared decision-making, and apply geriatric and palliative principles in the ICU environment.

## Ethics Approval

Approved by the Hospital Moinhos de Vento Research Ethics Committee (CAAE 5.565.048).

## References

[1] Bagshaw SM, Webb SA, Delaney A, George C, Pilcher D, Hart GK (2009). Very old patients admitted to intensive care in Australia and New Zealand: a multi-centre cohort analysis. Crit Care.

[2] Andersen FH, Flaatten H, Klepstad P, Romild U, Kvåle R (2015). Long-term survival and quality of life after intensive care for patients 80 years of age or older. Ann Intensive Care.

[3] Guidet B, Leblanc G, Simon T, Woimant M, Quenot JP, Ganansia O (2017). ICE-CUB 2 Study Network. Effect of systematic intensive care unit triage on long-term mortality among critically ill elderly patients in France: a randomized clinical trial. JAMA.

[4] Ehlenbach WJ, Barnato AE, Curtis JR, Kreuter W, Koepsell TD, Deyo RA (2009). Epidemiologic study of in-hospital cardiopulmonary resuscitation in the elderly. N Engl J Med.

[5] Ferrand E, Robert R, Ingrand P, Lemaire F (2001). French LATAREA Group. Withholding and withdrawal of life support in intensive-care units in France: a prospective survey. Lancet.

[6] Thietart S, Boumendil A, Pateron D, Guidet B, Vallet H (2022). ICE-CUB2 Study Network. Impact on 6-month outcomes of hospital trajectory in critically ill older patients: analysis of the ICE-CUB2 clinical trial. Ann Intensive Care.

[7] Curtis JR, White DB (2008). Practical guidance for evidence-based ICU family conferences. Chest.

[8] Ramos JG, Bautista MM, Calazans R, Melo L, Teixeira C (2024). Revolutionizing care: unleashing the power of comprehensive geriatric assessment in tailoring treatment for frail postintensive care patients. Crit Care Sci.

[9] Guidet B, Vallet H, Flaatten H, Joynt G, Bagshaw SM, Leaver SK (2024). The trajectory of very old critically ill patients. Intensive Care Med.

[10] Detering KM, Hancock AD, Reade MC, Silvester W (2010). The impact of advance care planning on end of life care in elderly patients: randomized controlled trial. BMJ.

